# HTR1A Inhibits the Progression of Triple‐Negative Breast Cancer via TGF‐*β* Canonical and Noncanonical Pathways

**DOI:** 10.1002/advs.202105672

**Published:** 2022-02-24

**Authors:** Qiqi Liu, Hefen Sun, Yang Liu, Xuan Li, Baojin Xu, Liangdong Li, Wei Jin

**Affiliations:** ^1^ Department of Breast Surgery Key Laboratory of Breast Cancer in Shanghai Fudan University Shanghai Cancer Center Shanghai 200032 China; ^2^ Department of Oncology Shanghai Medical College Fudan University Shanghai 200032 China

**Keywords:** HTR1A, metastasis, triple negative breast cancer, T*β*RII

## Abstract

Triple‐negative breast cancer is the most aggressive subtype of breast cancer and the incidence of depression in breast cancer patients is high, which leading to worse survival and increased risk of recurrence. The effect of antidepressants on breast cancer patients remains contradictory, which might be due to variations in antidepression targets. Therefore, there is significant value to explore the antitumor potential of antidepressants and discover new therapeutic targets for breast patients. The authors screen antidepressant‐related oncogenes or suppressors by using siRNAs. After combining functional experiments with online database analysis, 5‐hydroxytryptamine receptor 1A (HTR1A is selected with antitumor potential in breast cancer cells in vivo and in vitro. RNA‐seq analysis and coimmunoprecipitation assays indicate that HTR1A interacts with TRIM21 and PSMD7 to inhibit the degradation of T*β*RII through the ubiquitin‐proteasome pathway, thereby inhibiting the transforming growth factor‐*β* (TGF‐β) canonical and noncanonical pathway. In addition, HTR1A is an independent predictive factor for breast cancer patients. The combined treatment of HTR1A agonists with demethylation drugs may significantly improve patient survival. It is of great significance to clarify the function and mechanism of the depression‐related gene HTR1A in breast cancer, which might provide a new approach for triple‐negative breast cancer patients.

## Graphical abstract

This study confirms that 5‐hydroxytryptamine receptor 1A significantly inhibits the development of triple‐negative breast cancer (TNBC) cells in vivo and in vitro via downregulating the TGF‐*β* canonical and noncanonical pathways, which is an independent prognostic factor for TNBC patients. This study will help to clarify the role of antidepressants and discover more antitumor targets for TNBCcancer.

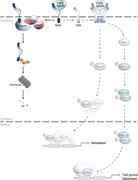

## 1 Introduction

Breast cancer is the most common malignant tumor with the highest incidence and the second highest mortality rate. In 2020, ≈685 000 women died of breast cancer worldwide, accounting for 15% of cancer‐related deaths in women.^[^ ^1^, ^2^ ^]^ Approximately 20% of breast cancer patients have recurrence and metastasis, causing ≈90% of breast cancer patients to die.^[^ ^3^ ^]^ Triple‐negative breast cancer (TNBC) is the most aggressive subtype of breast cancer, with the most aggressive phenotype and higher metastasis and recurrence rates than other subtypes. Due to the negative expression of estrogen receptor (ER), progesterone receptor (PR) and HER2 in TNBC patients, treatment options are limited. The prognosis of TNBC patients is poor, and the 5‐year disease‐free survival rate is only ≈80%.^[^ ^4^, ^5^ ^]^ Therefore, it is urgent to discover novel therapeutic targets for TNBC patients.

Due to younger diagnosis age, lack of social support, physical changes and clinical symptoms caused by treatment, the depression incidence in breast cancer patients is higher than that of other malignant tumor patients.^[^ ^6^, ^7^ ^]^ Statistics showed that 32.8% of breast cancer patients had depressive symptoms, and 42% of patients with recurrent breast cancer were diagnosed with depression, leading to increased mortality, worse clinical prognosis and quality of life.^[^ ^8^, ^9^ ^]^ A meta‐analysis showed that depression increases the overall risk of death in breast cancer patients by 30%, breast cancer‐specific mortality by 29%, and the risk of recurrence by 24%.^[^ ^10^ ^]^ Antidepressants are widely used at present, and previous studies have indicated that antidepressants may play a key role in tumorigenesis and development. However, depression is undertreated among breast cancer patients, with only half receiving antidepressant treatment.^[^ ^11^, ^12^ ^]^ In addition, epidemiological data have shown inconsistent results for correlations between different antidepressants and breast cancer risk. We speculated that the conflicting conclusions might be due to the heterogeneity of the different targets of antidepressants.

To screen the function of antidepressant‐related genes in TNBC, we knockdown them with effective siRNAs. After combining functional experiments with online database analysis, HTR1A (5‐hydroxytryptamine receptor 1A) was selected with antitumor potential in breast cancer cells. HTR1A belongs to the G protein‐coupled receptor family (GPCRs) and is one of the most widely expressed and abundant subtypes of serotonin receptors.^[^ ^13^ ^]^ Binding with ligand 5‐HT (5‐hydroxytryptamine) could result in conformational changes and signaling pathway activation. HTR1A also plays crucial roles in depression,^[^ ^14^ ^]^ schizophrenia,^[^ ^15^, ^16^ ^]^ wound healing,^[^ ^17^ ^]^ and attention deficit hyperactivity disorder.^[^ ^18^ ^]^ However, researches focusing on the function of HTR1A in tumors are limited, especially in TNBC. Online database analysis showed that HTR1A was relative lower in TNBC than non‐TNBC tissues. Therefore, it is of great importance to clarify the function and mechanism of HTR1A in TNBC, which could lead to more therapeutic targets for TNBC and improve the prognosis of patients.

## 2 Results

### 2.1 Depression‐Related Genes that Play Roles in Breast Cancer

**Figure 1:**
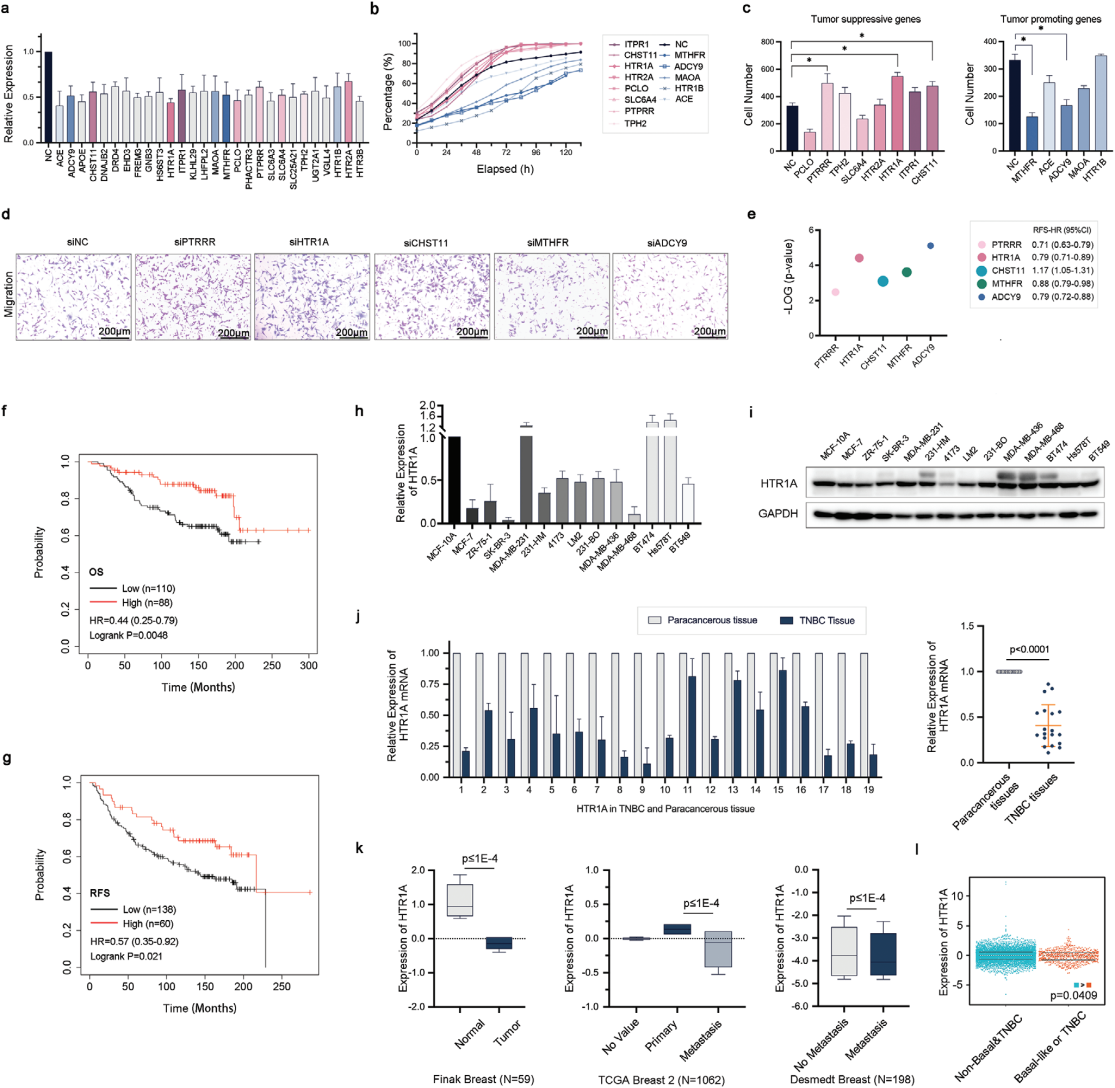
Depression‐related genes that play roles in breast cancer. a) The expression of 28 depression‐related genes after silenced by siRNA in LM2 cells. b,c) The proliferation and migration ability of breast cancer cells with specific gene silenced. The pink graph represents the tumor suppressive genes, while the blue graph represents the tumor promoting genes. d) The representative image of migration of HTR1A, PTRRR, CHST11, MTHFR, and ADCY9. e) The recurrence free survival (RFS) probability and hazard ratio (HR) of HTR1A, PTRRR, CHST11, MTHFR, and ADCY9 in Kaplan–Meier plotter database. f,g) The Kaplan–Meier plotter database showed the OS and RFS with the expression of HTR1A in breast cancer patients.h,i) Real‐time PCR and western blotting showed the mRNA and protein expression of HTR1A in breast cancer cell lines, respectively. j) The expression of HTR1A in TNBC tissues compared with their paracancerous tissues. k) The Oncomine database showed the expression of HTR1A in normal and tumor tissue(left), primary and metastatic breast cancer (middle), and non metastasis and metastasis cancer (right ). l) The *bc‐GenExMiner v4.8* database indicated the expression of HTR1A in TNBC and non‐TNBC tissues.

The corresponding expression of 28 depression‐related genes, which were selected by national center for biotechnology information (NCBI) and Gene Cards, was silenced in the breast cancer cell line MDA‐MB‐231 LM2 (LM2) by siRNAs and their knockdown efficacy was confirmed (**Figure** **1**a). Then the proliferation and metastasis ability of breast cancer cells were analyzed (Figure 1b,c). We found that knockdown HTR1A, PTRRR, and CHST11 can enhance while MTHFR and ADCY9 can reduce cell migration (Figure 1d). Next, we analyzed the survival benefits in the Kaplan–Meier plotter online database and found that HTR1A had the most significant survival benefit (Figure 1e and Figure S1, Supporting Information) and high expression of HTR1A had longer overall survival (OS) and recurrence‐free survival (RFS, Figure 1f,g, *p* < 0.05, respectively). Real‐time polymerase chain reaction (PCR) and western blotting results showed that HTR1A was generally higher in immortalized breast epithelial cells and in breast cancer cell lines with lower metastatic potential than that in triple‐negative breast cancer cell lines with high metastatic potential (Figure 1h,i). In addition, paired TNBC and paracancerous tissue showed that the mRNA expression level of HTR1A in breast cancer tissues was significantly lower than that in normal breast tissues (Figure 1j, *p* < 0.0001). The *Oncomine* database indicated that the expression level of HTR1A in metastatic breast cancer was lower than that in primary breast cancer (Figure 1k, *p* ≤ 1 ×10^−4^, fold change ≥ 2, respectively). The *bc‐GenExMiner* online database^[^ ^19^ ^]^ showed that HTR1A expression is significantly higher in non‐TNBC (or nonbasal like) tissues, compared with that in TNBC tissues (basal like) (Figure 1l, *p* = 0.0409). The results indicated that HTR1A may function as a tumor suppressor.

### 2.2 HTR1A Inhibits Cancer Cell Proliferation and Metastasis In Vivo and In Vitro

**Figure 2:**
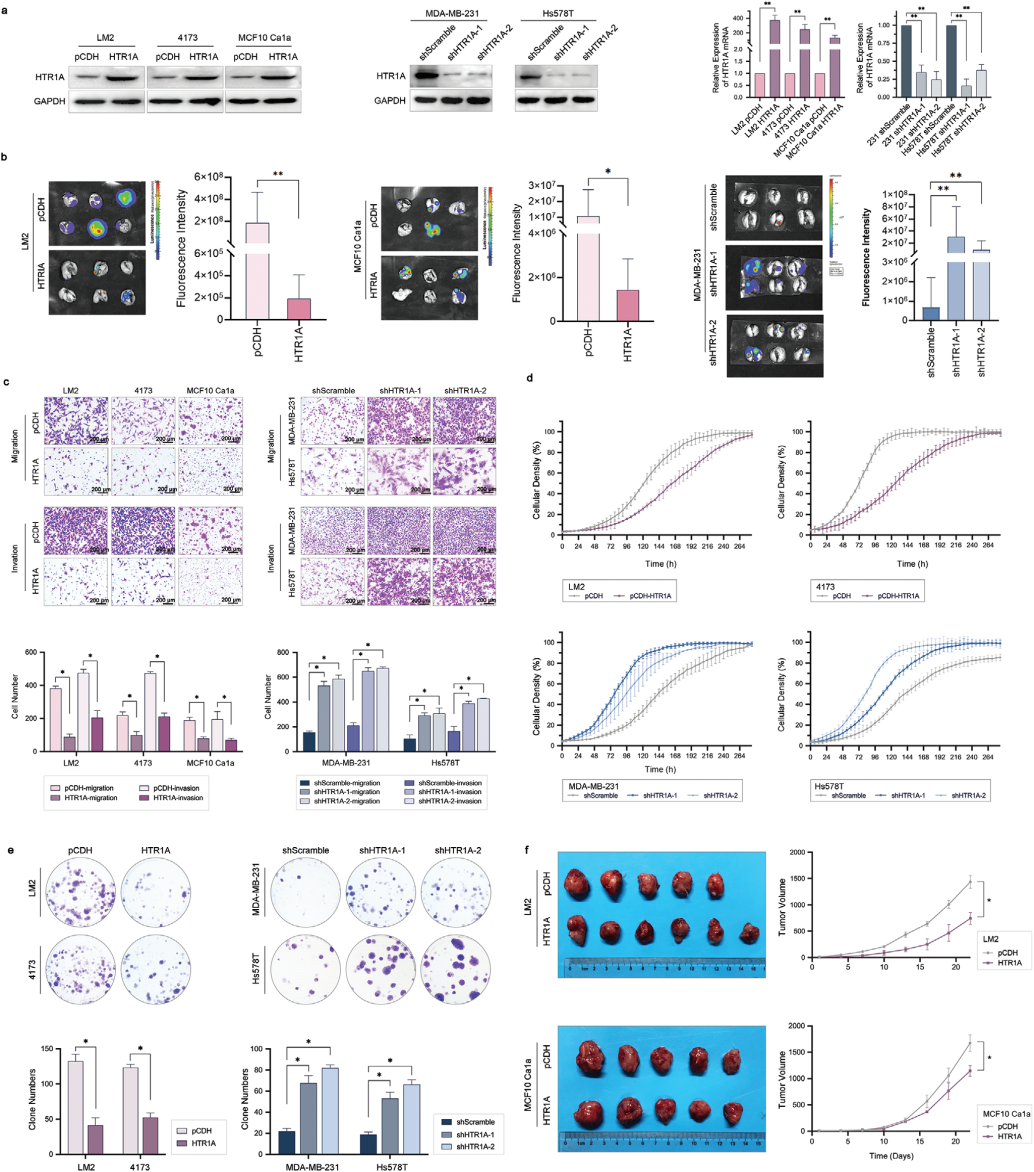
HTR1A inhibits cancer cell growth and metastasis in vivo and in vitro. a) HTR1A overexpressing and knockdown cell lines were constructed. b) Bioluminescent images (BLI) of mice with HTR1A‐overexpressing LM2, MCF10‐Ca1a, and MDA‐MB‐231 HTR1A‐knockdown cells. c) The migration and invasion ability in HTR1A overexpression and knockdown cells. d) The proliferation of cells with HTR1A overexpression and knockdown cells. e) Cloning formation assay with HTR1A overexpression and knockdown cells. f) HTR1A significantly inhibited the tumor growth of mouse xenograft models injected with LM2 and Ca1a (n = 6). Data are means ± SD,*p < 0.05 and **p < 0.01.

To explore the function of HTR1A in triple‐negative breast cancer, we constructed stable overexpression cell lines with LM2, 4173, and MCF10 Ca1a cells and knockdown with MDA‐MB‐231 and Hs578T cells (**Figure** **2**a). The results showed that HTR1A overexpression significantly inhibited lung metastasis in vivo. In contrast, HTR1A knockdown increased lung metastasis in mice (Figure 2b). Furthermore, Transwell assay showed that HTR1A overexpression significantly suppressed the migration and invasion ability of LM2, 4173 and MCF10 Ca1a cells, while knockdown significantly promoted the migration and invasion of MDA‐MB‐231 and Hs578T cells (Figure 2c). The results were also confirmed by the wound healing assay (Figure S2, Supporting Information). These observations indicate that HTR1A can significantly inhibit breast cancer cell metastasis both in vitro and in vivo.

We also found that HTR1A overexpression significantly inhibited the proliferation of breast cancer cells, while knockdown showed the opposite trend (Figure 2d). In addition, a cloning assay showed that HTR1A significantly inhibited the clone formation ability of breast cancer cells (Figure 2e). In addition, TNBC xenograft models constructed with LM2 and MCF10 Ca1a showed that HTR1A could significantly inhibit the tumorigenicity of breast cancer cells in vivo (Figure 2f).

### 2.3 HTR1A Inhibits the TGF‐*β*/T*β*RII/Smad Canonical Pathway

**Figure 3:**
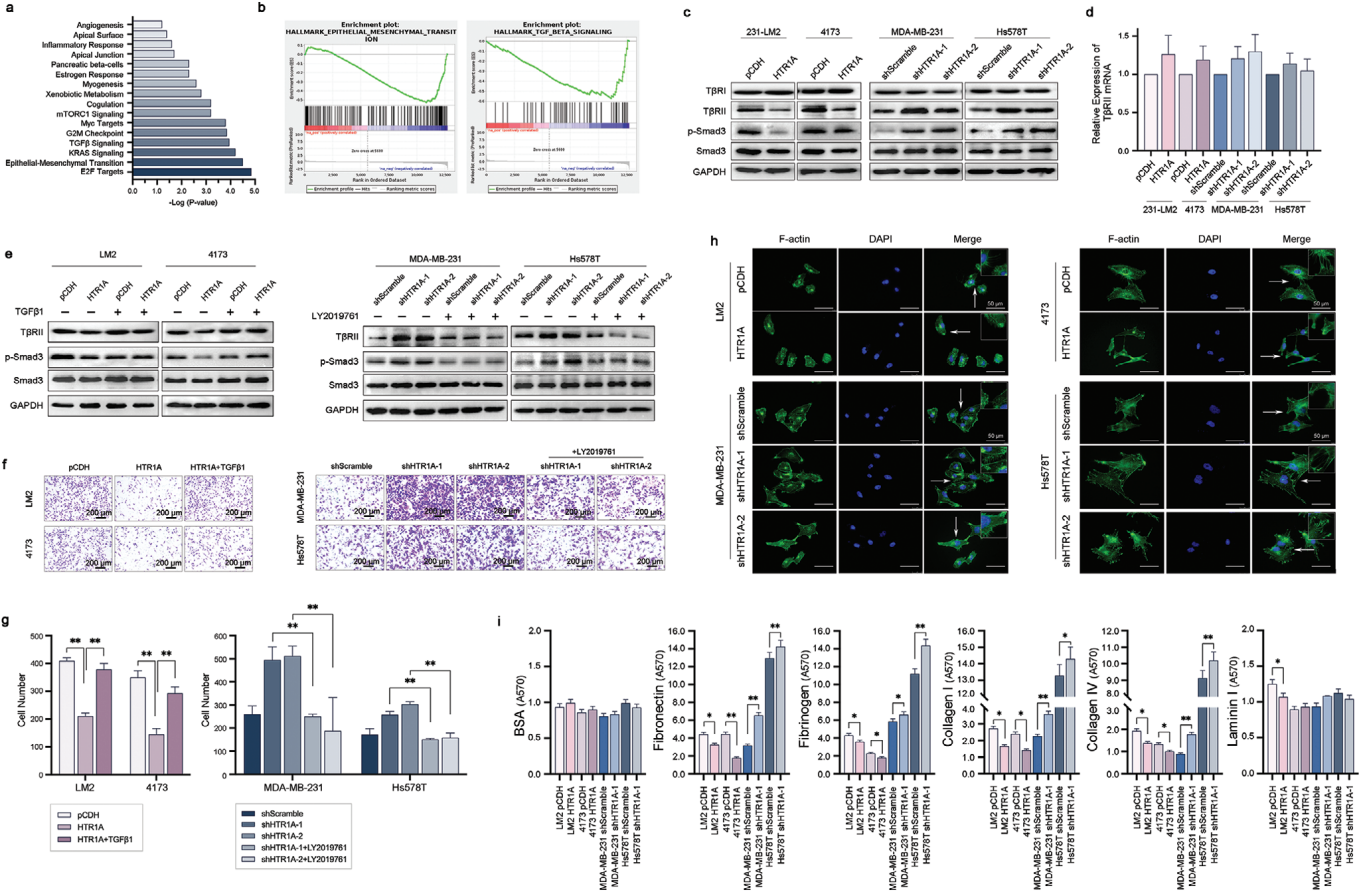
HTR1A inhibits the TGF‐*β*/T*β*RII/Smad canonical pathway. a,b) RNA‐seq of HTR1A overexpressing cells were analyzed by KEGG and GSEA, while TGF‐*β* and EMT pathway were enriched. c,d) HTR1A significantly inhibited the expression of T*β*RII and p‐Smad3 proteins, while no significant change was shown in the mRNA level. e–g) TGF‐*β*1 and LY2109761 rescued the signaling pathway and migration ability of breast cancer cells mediated by HTR1A. h) F‐actin staining of HTR1A overexpressing and knockdown cells. i) Cell adhesion analyzes of HTR1A overexpressing and knockdown cells. Data are means ± SD, *n* = 3. *p < 0.05 and **p < 0.01.

To further explore the specific mechanism underlying the effect of HTR1A on breast cancer, RNA‐seq was performed in LM2 pCDH and HTR1A‐pCDH, 4173 pCDH and HTR1A‐pCDH stable overexpression cells, and the transforming growth factor‐β (TGF‐β) pathway and epithelial to mesenchymal transition (EMT) pathway were enriched by kyoto encyclopedia of genes and genomes (KEGG) and gene set enrichment analysis (GSEA), **Figure** **3**a,b). Western blotting showed that HTR1A overexpression significantly inhibited the expression of T*β*RII and p‐Smad3 proteins, while T*β*RI showed tiny changes in LM2 and 4173 cells (Figure 3c). Notably, Real‐time PCR showed no significant change in the mRNA expression level of T*β*RII (Figure 3d). Furthermore, treatment with the T*β*RII agonist TGF‐*β*1 rescued the reduced expression of T*β*RII and p‐Smad3 as well as the migration ability of breast cancer cells mediated by HTR1A overexpression. Treatment with the T*β*RII inhibitor LY2109761 also rescued the effect of HTR1A knockdown on T*β*RII/p‐Smad3 expression and the migration ability of breast cancer cells (Figure 3e–g). The results showed that HTR1A inhibited breast cancer metastasis by impeding the TGF‐*β* signaling pathway.

Immunofluorescence staining of F‐actin showed that HTR1A inhibited the formation of pseudopodia and resulted in a round and blunt appearance and decreased the metastatic potential of breast cancer cells (Figure 3h). Cell adhesion analysis showed that HTR1A significantly inhibited the expression of Fibronectin 1 (FN‐1), Fibrinogen, Collagen I, Collagen IV and Laminin I with bovine serum albumin (BSA) as a negative control, while HTR1A knockdown showed the opposite trend (Figure 3i).

### 2.4 HTR1A Inhibits the TGF‐*β*/T*β*RII/MEK/ERK/c‐Myc Noncanonical Pathway

**Figure 4:**
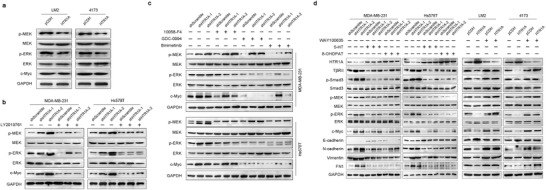
HTR1A inhibits the TGF‐*β*/T*β*RII/MEK/ERK/c‐Myc noncanonical pathway. a) HTR1A significantly inhibited the phosphorylation levels of MEK and ERK as well as the expression level of c‐Myc. b) LY2109761 could rescue the noncanonical MEK/ERK/c‐Myc signaling pathway in HTR1A stable knockdown cell lines. c) The effect of 10058‐F4, GDC‐0994, and Binimetinib treatment on the activation level of MEK/ERK/c‐Myc signaling pathway in HTR1A knockdown cell lines. d) The effect of WAY100635, 5‐HT, and 8‐OHDPAT on TGF‐*β* canonical and noncanonical pathways in HTR1A stable cell lines.

In addition to the canonical Smad pathway, T*β*RII can also activate noncanonical signaling pathways such as mitogen‐activated protein kinase (MAPK) and PI3K (phosphoinositide‐3‐kinase)‐serine/threonine kinase (AKT). Mitogen‐activated protein kinase kinase/mitogen‐activated protein kinase (MEK/ERK) is the main MAPK signal transduction pathway and plays a key role in tumorigenesis. To further verify whether HTR1A regulates the noncanonical signaling pathway mediated by TGF‐*β*/T*β*RII, we detected the key proteins in the signaling pathway. The results showed that HTR1A significantly inhibited the phosphorylation levels of MEK and ERK as well as the expression level of c‐Myc (**Figure** **4**a). The T*β*RII antagonist LY2109761 rescued the phosphorylation and expression levels of key molecules in the T*β*RII downstream noncanonical MEK/ERK/c‐Myc signaling pathway in HTR1A stable knockdown cells (Figure 4b). Furthermore, the phosphorylation levels of MEK and ERK were not affected by 10058‐F4 c‐Myc inhibitor, while GDC‐0994 ERK inhibitor did not affect the phosphorylation level of MEK. However, the activation level of the MEK/ERK/c‐Myc signaling pathway was significantly inhibited by binimetinib MEK inhibitor in HTR1A knockdown cell lines (Figure 4c). These results verified that HTR1A regulates the function of breast cancer by inhibiting the TGF‐*β*/T*β*RII/MEK/ERK/c‐Myc noncanonical signaling pathway. HTR1A ligand 5‐HT and agonist 8‐hydroxy‐2‐(di‐n‐propylamino)tetralin (8‐OHDPAT) were used to verify the effect of HTR1A. The results showed that 5‐HT inhibited the TGF‐*β*/T*β*RII/Smad canonical pathway and TGF‐*β*/T*β*RII/MEK/ERK/c‐Myc noncanonical pathway, while WAY100635 T*β*RII antagonist inhibited the Smad canonical pathway and MEK/ERK/c‐Myc noncanonical pathway mediated by HTR1A overexpression. The results suggest that HTR1A simultaneously inhibits the Smad canonical signaling pathway and MEK/ERK/c‐Myc noncanonical signaling pathway downstream of T*β*RII. In addition, western blotting showed that HTR1A downregulated mesenchymal cell markers, including N‐cadherin, FN‐1 and Vimentin, while upregulating the epithelial cell marker E‐cadherin. HTR1A antagonist WAY100635 treatment suppressed the activation of the EMT signaling pathway, while HTR1A ligand 5‐HT or agonist 8‐OHDPAT led to the opposite effect (Figure 4d).

### 2.5 HTR1A Interacts with TRIM21 and PSMD7 to Inhibit the Degradation of T*β*RII

**Figure 5:**
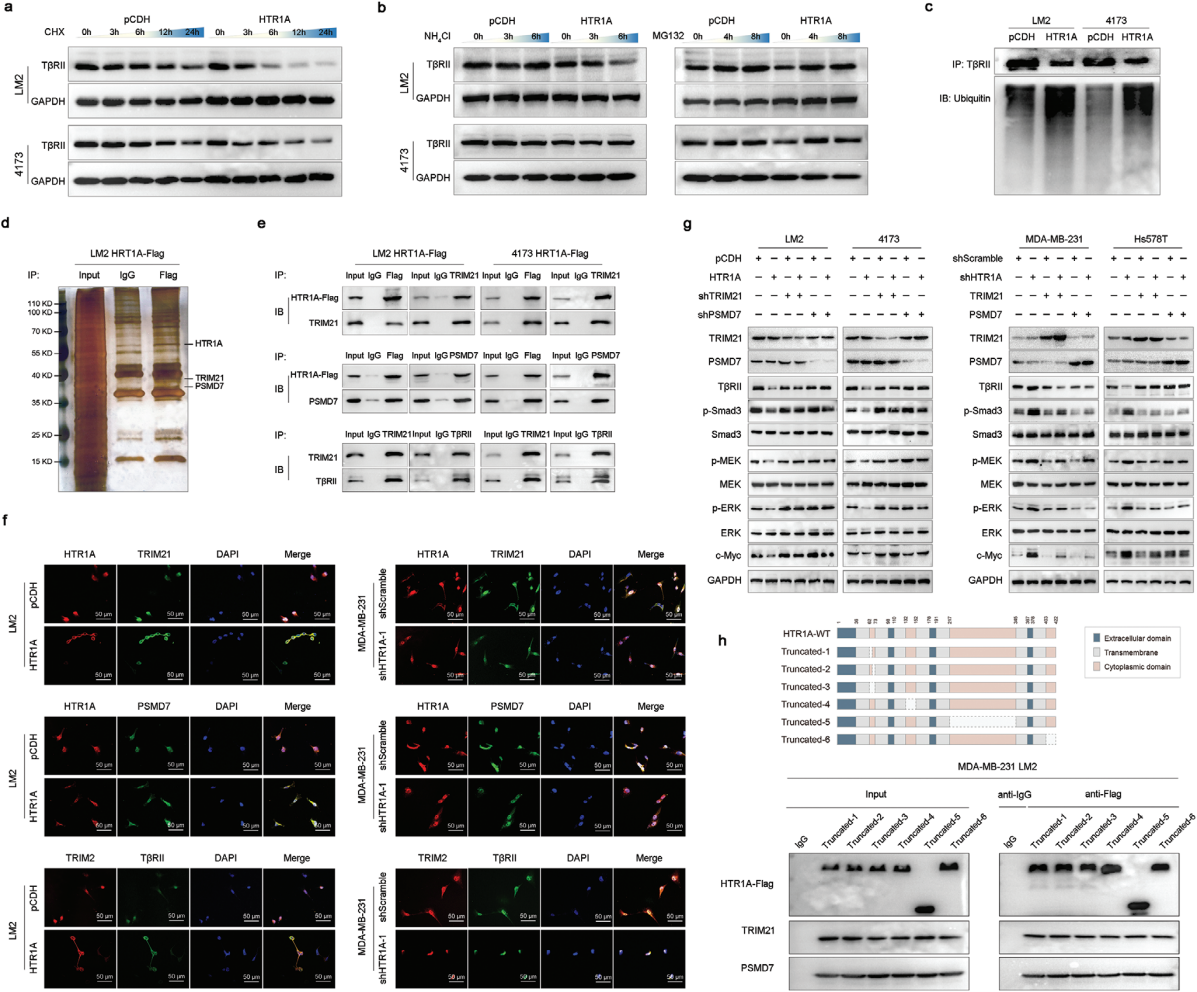
HTR1A interact TRIM21 and PSMD7 to inhibit the degradation of T*β*RII. a) The half‐life of T*β*RII was shorter in HTR1A overexpression cells treated with CHX. b) The expression level of T*β*RII was effectively rescued after MG132 treatment. c) The T*β*RII‐binding ubiquitin level in HTR1A overexpression cells was significantly higher. d) The protein bands interacted with HTR1A was detected by co‐IP and silver staining specific experiments. e,f) The interaction between HTR1A and proteins were verified by Co‐IP and confocal microscopy. g) TRIM21 and PSMD7 were crucial in HTR1A‐mediated TGF‐*β* pathway. h) The interaction of HTR1A with TRIM21 and PSMD7 was not affected by short‐truncated mutations of HTR1A intracellular regions.

Previous results showed no significant change in the mRNA level of T*β*RII in HTR1A stable overexpression and knockdown cells. We speculated that HTR1A regulates breast cancer progression and metastasis by promoting the protein degradation of T*β*RII. To confirm this, the T*β*RII expression level was assessed at different time points, while cycloheximide (CHX) was used to inhibit protein biosynthesis. The results showed that after CHX treatment, the expression of T*β*RII protein decreased faster and the half‐life was shorter in HTR1A‐overexpressing cells (**Figure** **5**a). Furthermore, the autophagy lysosome inhibitor ammonium chloride (NH_4_Cl, 10 × 10^−6^ m) and ubiquitin‐proteasome inhibitor (MG132, 10 × 10^−6^ m) were used to clarify the specific degradation pathway of T*β*RII. The results showed that the T*β*RII expression level in HTR1A‐overexpressing cells treated with NH_4_C1 for 4 and 8 h was still lower, while the expression level of T*β*RII was effectively rescued 4 and 8 h after MG132 treatment (Figure 5b). In addition, immunoprecipitation (IP) assays showed that the T*β*RII‐binding ubiquitin level in HTR1A‐overexpressing cells was significantly higher than that in the control group (Figure 5c). The above results suggest that HTR1A promotes the ubiquitin‐proteasome degradation pathway of T*β*RII.

Furthermore, co‐IP and silver staining specific experiments revealed that protein bands interacted with HTR1A at 48 and 37 kD, which were detected by IP in combination with mass spectrometry (IP‐MS, Figure 5d). Several of the enriched proteins were related to the ubiquitin‐proteasome degradation pathway, among which TRIM21 and PSMD7 were detected (Table S3, Supporting Information). Co‐IP verified the interaction between HTR1A and TRIM21, HTR1A and PSMD7, and TRIM21 and T*β*RII (Figure 5e). In addition, confocal microscopy showed colocalization between HTR1A and TRIM21, as well as HTR1A and PSMD7, which was significantly enhanced in HTR1A‐overexpressing cells and diminished in HTR1A knockdown cell lines. TRIM21 and T*β*RII also showed colocalization, which further confirmed that TRIM21 was involved in the ubiquitin‐proteasome degradation pathway of the T*β*RII protein (Figure 5f). Furthermore, HTR1A‐mediated downregulation of the TGF‐*β*/T*β*RII/Smad canonical pathway and TGF‐*β*/T*β*RII/MEK/ERK/c‐Myc noncanonical signaling pathway was significantly rescued after TRIM21 and PSMD7 knockdown. TRIM21 and PSMD7 overexpression in HTR1A stable knockdown cell lines led to the opposite effect (Figure 5g). Therefore, TRIM21 and PSMD7 play significant roles in the antitumor effect of HTR1A through the TGF‐*β* pathway in breast cancer cells.

As a seven‐transmembrane‐spanning receptor, HTR1A has four intracellular protein regions. To explore the specific functional domain of HTR1A binding with TRIM21 and PSMD7, seven mutant clones were constructed. The interaction between HTR1A mutant clones and interacting proteins was detected by Co‐IP. The results showed that the interaction of HTR1A with TRIM21 and PSMD7 was not affected by any short‐truncated mutations of HTR1A intracellular regions (Figure 5h). Thus, we speculate that the subunits and oligomeric proteins of HTR1A form a complex intracellular spatial domain, which may bind with TRIM21 and PSMD7 at multiple sites. The exact functional binding site of HTR1A remains unknown and needs additional research in the future.

### 2.6 Correlation between Methylation of the HTR1A Promoter and Gene Expression

**Figure 6:**
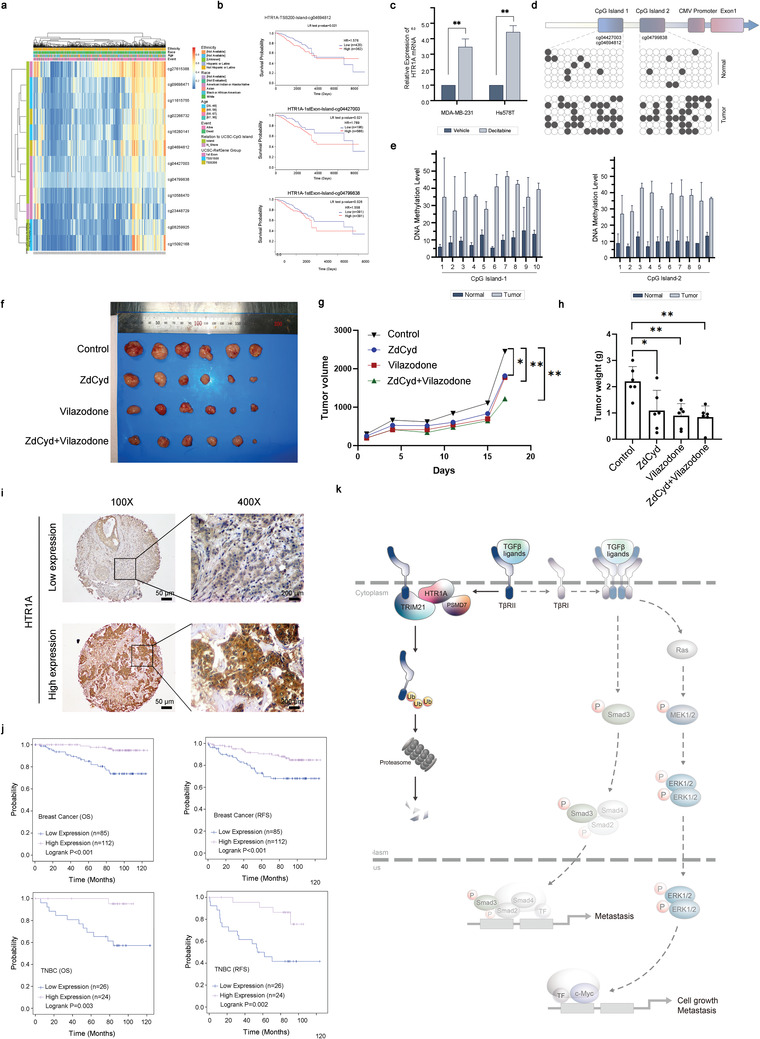
The correlation between HTR1A expression and survival. a,b) High methylation levels of CpG site cg04694812, cg04427003, and cg04799838 in HTR1A were significantly correlated with shorter OS of breast cancer patients c) The expression of HTR1A was significantly upregulated after decitabine treatment. d,e) HTR1A CpG site in breast cancer tissues showed significantly higher methylation levels. f–h) The antitumor effect of ZdCyd and vilazodone in mouse xenograft models of MDA‐MB‐231 LM2. i,j) The correlation between HTR1A expression and survival was analyzed by the Cox proportional hazard regression model. k) Model of the mechanism of HTR1A in triple‐negative breast cancer. *p < 0.05 and **p < 0.01.

Previous studies discovered that HTR1A expression was correlated with the level of promoter methylation. The MethSurv database showed that high methylation levels of cg04694812, cg04427003, and cg04799838 in HTR1A were significantly correlated with shorter OS in breast cancer patients (**Figure** **6**a,b). Furthermore, the demethylation drug 5‐aza‐2′‐deoxycytidine (1 × 10^−6^ m, 5‐AZA‐CdR, ZdCyd, decitabine) was utilized for 7 d, after which the expression of HTR1A was significantly upregulated in breast cancer cells (Figure 6c, all p < 0.01). In addition, bisulfite sequencing of specific CpG sites was performed on DNA samples from 19 pairs of triple‐negative breast cancer and paracancerous tissues. The results showed that compared with adjacent normal breast tissues, HTR1A CpG sites cg04694812, cg04427003, and cg04799838 in breast cancer tissues showed significantly higher methylation levels (Figure 6d,e, all *p* < 0.05). Notably, previous results showed that the expression level of HTR1A in breast cancer tissues was significantly lower than that in paracancerous tissues, indicating that there was a significant negative correlation between HTR1A expression and the CpG site methylation level. Inhibition of promoter methylation at specific CpG sites led to upregulation of HTR1A expression.

In addition, to explore the antitumor effect of the demethylation drug combining with the HTR1A agonist, we first screened several of HTR1A‐agonist drugs and investigated their inhibition rate on LM2 breast cancer cells. Among all the agonists, vilazodone and flibanserin were shown with the most significant inhibition rate (Figure S3a, Supporting Information). Further, we established xenograft models of LM2 cells in BALB/c (nu/nu) mice. The results showed that both vilazodone and flibanserin treatment exerted antitumor effects (Figure 6f–h and Figure S3b, Supporting Information). Then we found that the tumor weights in mice treated with one of demethylation drugs 5‐aza‐2’‐deoxycytidine (ZdCyd) or vilazodone were significantly lower than those in the control group (Figure 6h). In addition, 5‐aza‐2’‐deoxycytidine combined vilazodone was the most efficient at impeding tumor growth of mouse xenograft models (Figure 6f–h).

### 2.7 The Correlation between HTR1A Expression and Survival

To explore the relationship between HTR1A and survival in breast cancer patients, 197 breast cancer patients were analyzed by immunohistochemistry (IHC) staining and the Cox proportional hazard regression model (**Table** **1** and Figure 6i). The results showed significantly shorter OS and RFS in TNBC patients with lower expression levels of HTR1A. In addition, all breast cancer patients with higher expression of HTR1A showed significantly longer OS and RFS. However, there was no significant correlation between HTR1A and survival in HER2‐overexpressing subtype and luminal subtype patients (Figure 6j).

**Table 1:** The relationship between clinical characteristics and HTR1A expression level in breast cancer patients

Variables		Low expression		High expression		*p‐*value
		*n* = 85	%	*n* = 112	%	
Age	<49	45	52.94	67	59.82	0.401
	≥50	40	47.06	45	40.18	
Menstrual status	Premenopause	50	58.82	69	61.61	0.692
	Postmenopause	35	41.18	43	38.39	
Grade	I	2	2.35	3	2.68	0.916
	II	58	97.65	79	70.54	
	III	25	29.41	30	26.79	
Tumor size	≤2	44	51.76	54	48.21	0.776
(cm)	>2 and ≤5	38	44.71	52	46.43	
	>5	3	3.53	6	5.36	
TNM stage	I	28	32.94	36	32.14	0.537
	II	49	57.65	65	58.04	
	III	4	4.71	9	8.04	
	IV	4	4.71	2	1.79	
Lymph nodes	Negative	42	49.41	55	49.11	0.966
	Positive	43	50.59	57	50.89	
ER^a)^	Negative	52	61.18	58	51.79	0.189
	Positive	33	38.82	54	48.21	
PR^b)^	Negative	52	61.18	59	52.68	0.234
	Positive	33	38.82	53	47.32	
Her2^c)^ status	Negative	48	56.47	52	45.54	0.163
	Positive	37	43.53	60	54.46	

^a)^ ER: estrogen receptor

^b)^ PR: progesterone receptor

^c)^ Her2: epidermal growth factor receptor 2.

Furthermore, univariate and multivariate analyses showed that HTR1A expression was an independent prognostic factor of RFS in breast cancer patients and a high expression level suggested better survival. In addition, multivariate analysis showed that HER2, stage III and IV were also independent risk factors for breast cancer patients, while univariate analysis showed that ER and PR were independent risk factors. Other factors, such as age, menopausal status, histological grade, pathological type, tumor diameter, and lymph node metastasis status, showed no significant correlation with RFS in breast cancer patients (**Table** **2**).

**Table 2:** The univariate and multivariate analysis of clinicopathological features and recurrence‐free survival in breast cancer patients

Variables		Univariate analysis		Multivariate analysis	
		HR (95% CI^a)^)	*p* value	HR (95% CI)	*p‐*value
Age	<49	Ref.		Ref.	
	≥50	1.192 (0.617–2.302)	0.601	0.888 (0.386–2.043)	0.780
Menopause status	No	Ref.		Ref.	
	Yes	1.216 (0.627–2.359)	0.563	0.999 (0.423–2.359)	0.998
Grade	I	Ref.		Ref.	
	II	7996.83 (0.000–)	0.914	3419.004 (0–)	0.912
	III	10193.7 (0.000–)	0.912	3423.619 (0–)	0.912
TNM stage	I	Ref.		Ref.	
	II	1.736 (0.738–4.085)	0.206	1.834 (0.722–4.660)	0.202
	III	3.946 (1.251–12.45)	0.019	6.756 (1.854–24.63)	0.004
	IV	7.234 (1.868–28.02)	0.004	12.18 (2.808–52.80)	0.001
Tumor size (cm)	≤2	Ref.		Ref.	
	>2 and ≤5	1.115 (0.569–2.185)	0.751	1.108 (0.518–2.372)	0.791
	>5	1.703 (0.393–7.379)	0.476	2.208 (0.459–10.62)	0.323
LN status	Negative	Ref.		Ref.	
	Positive	1.030 (0.536–1.980)	0.929	1.074 (0.510–2.265)	0.850
ER^b)^	Negative	Ref.		Ref.	
	Positive	0.426 (0.206–0.884)	0.022	0.701 (0.192–2.558)	0.591
PR^c)^	Negative	Ref.		Ref.	
	Positive	0.466 (0.225–0.966)	0.040	0.333 (0.158–2.130)	0.412
Her2^d)^	Negative	Ref.		Ref.	
	Positive	2.217 (0.865–5.650)	0.098	3.003 (1.414–6.369)	0.004
HTR1A	Low	Ref.		Ref.	
	High	0.502 (0.247–1.021)	0.057	0.406 (0.198–0.832)	0.014

^a)^ CI: confidence interval

^b)^ ER: estrogen receptor

^c)^ PR: progesterone receptor

^d)^ Her2: epidermal growth factor receptor 2.

## 3 Discussion

Our study comprehensively focused on the antitumor potential of depression‐related genes and discovered HTR1A as a novel antitumor target in breast cancer. HTR1A was one of GPCRs and is one of the most widely expressed and abundant subtypes of serotonin receptors.^[^ ^13^ ^]^ HTR1A, binding with its ligand 5‐HT (also known as serotonin), resulted in conformational changes and signaling pathway activation, such as PI3K/Akt and MAPK/ERK pathways.^[^ ^20^, ^21^ ^]^ Studies regarding the function of HTR1A are mainly focused on the nervous system, such as regulating depression by regulating self‐tolerance by deregulating gene expression. HTR1A also plays crucial roles in schizophrenia,^[^ ^15^, ^16^ ^]^ wound healing,^[^ ^17^ ^]^ and attention deficit hyperactivity disorder.^[^ ^18^ ^]^ In addition, studies have shown that the HTR1A agonist sumatriptan inhibits insulin secretion^[^ ^22^ ^]^ and increases the risk of type I diabetes by enhancing the activity of cytotoxic T cells.^[^ ^23^ ^]^ However, research focusing on the role of HTR1A in tumors is limited. For example, HTR1A was reported to induce immune escape through autophagy in lung adenocarcinoma patients.^[^ ^24^ ^]^ A study of breast cancer patients found that HTR1A was relatively highly expressed on the membrane of cancer cells and in the cytoplasm of nonmalignant cells.^[^ ^25^ ^]^ In addition, the interaction between estrogen and the HTR1A ligand 5‐HT plays a regulatory role in breast cancer by inducing apoptosis, but the specific mechanism is not clear.^[^ ^26^ ^]^ Our study uncovered the functions and mechanisms of HTR1A in the occurrence and development of breast cancer, which is helpful to clarify the role of depression‐related genes in breast cancer and may identify more potential therapeutic targets for breast cancer.

Thus far, it is particularly urgent to find novel therapeutic drugs for TNBC patients due to their high relapse and metastasis rates. Furthermore, the incidence of depression in breast cancer patients is relatively higher, which may lead to worse survival; therefore, attention should be given to the patient depressive state. In addition to traditional monoamine oxidase inhibitors (MAOIs) and tricyclic and tetracyclic antidepressants (TcAs), the new generation of antidepressants, such as selective serotonin reuptake inhibitors (SSRIs), is also widely used in malignant tumor patients with higher specificity and fewer side effects. Evidence has shown that some antidepressants exert their antitumor effects by inducing tumor cell apoptosis,^[^ ^27^ ^]^ inhibiting tumor cell proliferation^[^ ^28^ ^]^ and angiogenesis,^[^ ^29^ ^]^ and enhancing antitumor immunity.^[^ ^30^ ^]^ MAOIs inhibit the differentiation of acute myeloid leukemia cells to exert an antitumor effect.^[^ ^31^ ^]^ TcAs inhibit the occurrence and development of malignant tumors, including lung cancer,^[^ ^32^, ^33^ ^]^ glioma,^[^ ^34^, ^35^ ^]^ liver cancer,^[^ ^36^ ^]^ lymphoma,^[^ ^27^ ^]^ and melanoma.^[^ ^37^ ^]^ SSRIs induce apoptosis of lymphoma cells,^[^ ^38^ ^]^ inhibit the proliferation of colon cancer cells,^[^ ^39^ ^]^ and enhance the antitumor immunity of melanoma.^[^ ^40^ ^]^ The third‐generation antipsychotic aripiprazole can increase chemosensitivity and induce apoptosis of breast cancer cells.^[^ ^41^, ^42^ ^]^ Histamine receptor agonists used in patients with refractory depression significantly improve the therapeutic response and prolong the survival time of breast cancer patients.^[^ ^43^ ^]^ These results suggest that antidepressants have antitumor potential. However, epidemiological data have also shown inconsistent results between antidepressants and breast cancer risk. Some studies have suggested that several antidepressants, including sertraline, paroxetine, and tricyclics, increase the risk of breast cancer after treatment for longer than 2 years.^[^ ^44^ ^]^ There is also a number of studies showing that antidepressants are not related to breast cancer risk or recurrence.^[^ ^45^, ^46^ ^]^ We speculated that the conflicting results of antidepressants on breast cancer patients may be caused by the heterogeneity of different antidepressant targets. Therefore, it is of great importance to clarify the function and mechanism of depression‐related genes, which may lead to more therapeutic targets for breast cancer patients.

TGF‐*β* is a polypeptide member of the transforming growth factor superfamily, which is widely involved in cancer cell growth, apoptosis, differentiation, and metastasis. Our study found for the first time that HTR1A simultaneously inhibits the TGF‐*β*/T*β*RII/Smad canonical signaling pathway and TGF‐*β*/T*β*RII/MEK/ERK/c‐Myc noncanonical signaling pathway, which play significant roles in cancer development. The Smad canonical pathway plays a key role in breast cancer metastasis by regulating EMT, resulting in cell polarity loss, cytoskeleton remodeling, and extracellular matrix degradation. In addition, the decrease in cell adhesion ability is the molecular basis of tumor cell infiltration and metastasis. EMT regulates breast cancer cells with an epithelial phenotype to lose their adhesion ability and acquire the migration ability of mesenchymal cells, thus promoting tumor metastasis. The MEK/ERK/c‐Myc noncanonical pathway is also crucial in regulating the metastatic ability of tumor cells.^[^ ^47^ ^]^ In addition, c‐Myc regulates the G1/S transition of the cell cycle through the cyclin‐dependent kinases (cyclin‐CDK) complex and plays important roles in tumor proliferation. HTR1A downregulation simultaneously activates these two pathways, thereby playing a key role in tumorigenesis and cancer development.^[^ ^48^ ^]^ The Model of the mechanism of HTR1A in triple‐negative breast cancer was shown in Figure 6k. Drugs targeting HTR1A and its ligands combined with these two signaling pathway inhibitors could have potential therapeutic value for TNBC patients.

The ubiquitin‐proteasome pathway is a crucial protein degradation regulatory system that participates in tumorigenesis through the accumulation of abnormal proteins.^[^ ^49^ ^]^ It is suggested that PSMD7 is the core of the proteasome 19S regulatory complex subunit and plays an important role in the presentation and degradation of ubiquitin substrates. TRIM21, as an E3 ubiquitin ligase, has been reported to be involved in the proliferation and metastasis of many malignant tumors by promoting polyubiquitin and proteasome degradation pathways.^[^ ^50^, ^51^ ^]^ In this study, it was found for the first time that HTR1A interacts with TRIM21 and PSMD7, promotes the ubiquitin‐proteasome degradation pathway of T*β*RII and inhibits downstream signaling pathways.

Furthermore, methylation modification is crucial in gene expression and stability, while aberrant promoter CpG methylation often leads to malignant transformation and tumor growth, which is a hot spot in tumor research.^[^ ^52^ ^]^ A variety of demethylation drugs have been developed, among which 5‐aza‐2’‐deoxycytidine, also known as decitabine, is the first food and drug administration (FDA)‐approved DNA demethylating agent used in malignant cancer patients.^[^ ^53^ ^]^ Studies have shown multiple CpG sites in the HTR1A promoter, which may regulate gene expression levels through methylation status.^[^ ^54^ ^]^ In our study, we found that the methylation level of the HTR1A promoter was significantly correlated with gene expression and patient survival. Therefore, the combined treatment of HTR1A agonists with demethylation drugs such as 5‐aza‐2’‐deoxycytidine may significantly improve the expression of HTR1A and the survival of patients, which might provide a new approach for the treatment of breast cancer patients.

This study identified the role of different antidepressant‐related genes in breast cancer, which is helpful to guide the use of antidepressants in breast cancer patients and improve the therapeutic effect and survival of breast cancer patients. This is the first study that comprehensively screened and verified the function of depression‐related genes in triple‐negative breast cancer, which is helpful to find more targets with antitumor potential for TNBC patients. In addition, antidepressants have been widely used in the clinic with clear mechanisms, toxicity and side effects, which is helpful for shortening the time from application to approval for therapies. Thus, antidepressants targeting HTR1A may have potential clinical value for TNBC patients in the future, with significant advantages compared with traditional drug development.

## 4 Conclusion 

In summary, this study confirmed that HTR1A inhibits the ubiquitin‐proteasome pathway of T*β*RII by interacting with TRIM21 and PSMD7, simultaneously downregulating the downstream Smad canonical pathway and MEK/ERK/c‐Myc noncanonical pathway, and thus inhibits cytoskeletal rearrangement and EMT to regulate the development of breast cancer. HTR1A expression can be upregulated by demethylase drugs, while the combination treatment of an HTR1A agonist with 5‐aza‐2′‐deoxycytidine may significantly increase the expression level of HTR1A and improve patient survival, which might provide a new approach for treating TNBC patients. The clinical analysis indicates HTR1A as an independent prognostic factor for patients with breast cancer and higher expression indicates better survival. This study will help to clarify the role of antidepressants in patients with breast cancer and may lead to the discovery of more antitumor targets for breast cancer.

## 5 Experimental Section

### Depression‐Related Genes Identification

Depression‐related genes that were reported in prior researches published in PUBMED were identified. The search terms included “meta‐analysis, reviews, association study, gene, depression, depressive disorder, major depression, bipolar disorder, bipolar affective disorder, manic depressive disorder, or manic depression.” Studies included in the research were based on the following criteria: a) published in a peer‐reviewed journal in English, b) provided the risk gene names for depression, and c) the genes determined significance at the *p* < 0.05 threshold. Four meta‐analyses studies and an article review regarding depressive disorder were identified.^[^ ^55^, ^56^, ^57^, ^58^, ^59^ ^]^ After further identifying the potential role of depression‐related genes in NCBI and GeneCards online database, a total of 28 genes that contribute to burden of depressive disorder were included in this research, including angiotensin I converting enzyme (ACE), ADCY9, APOE, CHST11, DNAJB2, DRD4, EHD3, FREM3, GNB3, HS6ST3, HTR1A, HTR1B, HTR2A, HTR3B, ITPR1, KLHL29, LHFPL2, MAOA, MTHFR, PCLO, PHACTR3, PTPRR, SLC6A3, SLC6A4, SLC25A21, TPH2, UGT2A1, and VGLL4.

### Cell Culture

Cancer cells were cultured in Dulbecco's modified Eagle's medium (DMEM, High Glucose, Gibco, USA) in tissue culture dishes (Thermo Fisher Scientific, Waltham, USA) or in Leibovitz's L‐15 medium (Gibco, USA) in tissue culture flasks (Thermo Fisher Scientific, USA) at 37 °C in a humidified incubator containing 95% air and 5% CO_2_. DMEM and L15 medium containing 10% heat‐inactivated fetal bovine serum (FBS, Gibco) and 1% penicillin‐streptomycin (Gibco, USA) were replaced every 2 d and examined periodically for mycoplasma contamination. Phosphate‐buffered saline (PBS) was obtained from Sigma‐Aldrich St. Louis, MO, USA.

### siRNA Transfection

Gene‐specific and corresponding negative control small interfering RNAs (siRNAs) were purchased from GenePharma (Shanghai, China). The siRNA target sequences are listed in Table S1 in the Supporting Information. According to the manufacturer's instructions, 3.0 × 10^5^ cells were inoculated in six‐well plates and three pairs of siRNA primers for each gene were transfected using Lipofectamine RNAiMAX transfection reagent in Opti‐MEMI serum reducing medium (Thermo Fisher Scientific, Waltham, MA, USA). Knockdown efficiency was validated by immunoblotting after 48 h of transfection.

### Cell Proliferation and Colony Formation Assays

Cell proliferation assays were conducted by IncuCyte ZOOM assay (Essen BioScience, Ann Arbor, MI, USA). For colony formation assays, 2 × 10^3^ cells were plated into six‐well plates in triplicate and cultured under normal conditions for 2 weeks. Colonies were fixed in methanol and stained with 0.5% crystal violet. Colonies with more than 50 cells were counted.

### Wound‐Healing, Cell Migration, and Invasion Assays

For wound‐healing assays, cells were seeded in six‐well plates and scratches were made by the AutoScratch Wound Making Tool (BioTek, Winooski, VT, USA). Cells were then washed with PBS and incubated in medium containing 0.1% FBS for the indicated times. For migration and invasion assays, 5 × 10^4^ cells in serum‐free medium were plated in the upper chambers coated with (invasion) or without (migration) growth factor‐reduced Matrigel (BD Biosciences, Franklin Lakes, NJ, USA). After 12 or 18 h, cells in the lower membranes of transwell chambers were fixed in methanol, stained with 0.5% crystal violet, and counted under a microscope.

### Cell Cycle

After counting by trypsin digestion, 3 × 10^4^ cells were inoculated into a six‐well plate in complete culture medium containing 2 × 10^−3^ m thymidine for 18 h and then replaced with complete medium. After 10 h, the medium was replaced with complete culture medium containing 2 × 10^−3^ m thymidine again for 16 h. The culture was terminated at 0, 6, 12, and 24 h after cell synchronization. Cells were fixed with precooled 70% ethanol at −20 °C overnight. The next day, the fixed cells were stained with 500 µL propidium iodide staining solution and analyzed by flow cytometry.

### Real‐time PCR

Total RNA was extracted with TRIzol (Invitrogen, USA) from cell lines and cDNA was then synthesized using a PrimeScript RT reagent kit (Perfect Real Time) (TAKARA, Japan) according to the manufacturer's protocol. Real‐time PCR was conducted using TB Green Premix Ex Taq (Tli RNaseH Plus, TaKaRa, Japan) on an applied biosystems (ABI) QuantStudio 6 Flex (Thermo Fisher Scientific, USA). The samples were amplified in three independent experiments. The primer sequences for each gene were as follows: T*β*RII, forward: 5′‐CGTGAAGAACGACCTAACCT‐3′, reverse: 5′‐TTTCCCAGAGCACCAGAGTT‐3′; HTR1A, forward: 5′‐GATCGAGGTGCACCGAGTGG‐3′, reverse: 5′‐CCCATGATGATGCCCAGCGT‐3′; and glyceraldehyde‐3‐phosphate dehydrogenase (GAPDH), forward: 5′‐GGAGCGAGATCCCTCCAAAAT‐3′, reverse: 5′‐GCTGTTGTCATACTTCTCATGG‐3. The 2^−ΔΔCt^ method was used to calculate relative gene expression, with GAPDH used as the reference gene for normalization.

### Western Blotting

Cell lysate was prepared in Pierce Tissue Protein Extraction Reagent (Thermo Fisher Scientific Inc.) with protease inhibitor and phosphatase inhibitors (Biomark), separated through electrophoresis, transferred onto polyvinylidene difluoride membranes, and then blocked with 5% nonfat milk (Sangon Biotech, Shanghai, China) in Tris‐buffered saline/0.1% Tween‐20 solution for 60 min at 37 °C. Next, membranes were incubated with primary antibodies overnight at 4 °C and the antibodies used are described in Table S2 in the Supporting Information. The membranes were subsequently incubated with appropriate secondary antibodies at room temperature for 1 h and visualized with chemiluminescence substrate (share‐bio, Shanghai, China). GAPDH (Proteintech, 1:20 000) served as the internal control for normalization. The band densities were quantified using ImageJ software (version 1.62, National Institutes of Health, Bethesda, MD, USA).

### Immunoprecipitation and Coimmunoprecipitation

For immunoprecipitation and coimmunoprecipitation analysis, 1 × 10^7^ cells were harvested with IP lysis buffer (20 × 10^−3^ m (2‐[4‐(2‐Hydroxyethyl)‐1‐piperazinyl] ethanesulfonic acid (HEPES), 150 × 10^−3^ m NaCl, 50 × 10^−3^ m KCl, 2 × 10^−3^ m MgCl_2_, 5 × 10^−3^ m ethylene diamine tetraacetic acid (EDTA) pH 8.0, 100 × 10^−3^ m NaF, 1% NP40, 10% glycerol). Cellular extracts were incubated with protein A/G magnetic beads (Sigma‐Aldrich, St. Louis, MO, USA), along with primary antibodies or control IgG in a rotating incubator overnight at 4 °C. The immunoprecipitates were washed three times with lysis buffer, eluted by boiling with sodium dodecyl sulfate (SDS) loading buffer for 10 min, and analyzed by western blotting. All antibodies used in this study are listed in Table S2 in the Supporting Information.

### Silver Staining and Mass Spectrometry

According to the protocols of the Pierce Silver Stain Kit (Thermo), gels were washed and fixed. Then, gels were incubated in Sensitizer Working Solution for exactly 1 min and then in Stain Working Solution for 30 min. After quickly washing gels with two changes of ultrapure water for 20 s each, Developer Working Solution was immediately added and incubated for 2–3 min until protein bands appeared. Finally, gels were washed briefly, and Stop Solution was replaced and incubated for 10 min. Then, the specific protein bands were subjected to tandem mass spectrometry (MS/MS) analysis by Shanghai Applied Protein Technology Co. Ltd. The resulting MS/MS data were processed using Proteome Discoverer 1.3. Mass error was set to 10 ppm for precursor ions and 0.02 Da for fragment ions. Carbamidomethyl on Cys was specified as a fixed modification and oxidation on Met was specified as a variable modification. Peptide confidence was set at high and the peptide ion score was set at >20.

### Immunofluorescent Staining

For immunofluorescent staining, cells were fixed with 4% paraformaldehyde, permeabilized in 0.1% Triton X‐100, and blocked with blocking buffer (Thermo). Then, the cells were incubated with the corresponding primary antibodies, washed three times using phosphate buffered saline and tween (PBST), and incubated with secondary antibodies conjugated with Alexa Fluor 647 (#ab150115, Abcam, Cambridge, MA, USA) or Alexa Fluor 488 (#ab150077). DNA was stained with fluoroshield mounting medium with 2‐(4‐Amidinophenyl)‐6‐indolecarbamidine dihydrochloride (DAPI), #ab104139). A Leica SP5 confocal laser scanning microscope (Leica Microsystems, Wetzlar, Germany) was used for imaging.

### Immunohistochemistry

IHC was performed employing the streptavidin‐peroxidase method. Briefly, formalin‐fixed and paraffin‐embedded tumor tissues were sliced into 4 µm thick sections and made into tissue microarrays. After dewaxing, rehydration, and citrate antigen retrieval, primary antibodies were applied to the sections and incubated overnight at 4 °C. Next, the slides were incubated with secondary antibodies (SP‐9000, ZSGB‐BIO, China) at 37 °C. Then, the slides were developed with 3,3’‐diaminobenzidine and counterstained with hematoxylin. Sections without primary antibodies were used as negative controls. The staining was scored as follows: 0, no staining; 1, <50% cells weakly stained; 2, ≥50% cells weakly stained; 3, <50% cells strongly stained; and 4, ≥50% cells strongly stained. The IHC staining was scored independently in double‐blindfolded manners by two researchers. Images of three representative fields were captured with an Olympus BX53 microscope (Olympus, Tokyo, Japan) at 200 × or 400 × magnification.

### Bisulfite Sequencing PCR

Genomic DNA was extracted from fresh breast cancer samples using a Genomic DNA Purification Kit (Qiagen, Hilden, Germany). The extracted DNA (1 µg) underwent bisulfite conversion using a sodium bisulfite procedure with the EZ DNA Methylation‐Gold Kit (Qiagen, Valencia, Canada) according to the manufacturer's protocol. Each of the DNA samples was amplified by PCR using methylation‐specific (MSP) PCR methylation primers, which were designed by MethPrimer2 as follows: CpG cg04694812/cg04799838, F‐primer: AGTTAATAAGATAATTGGTT, R‐primer: ACTTCCTACTTAAATCTCTA; CpG cg04799838, F‐primer: TATAGAGTATTTTTAGTTTC, R‐primer: AATAACCTACRACCTATTG. After visualization with ethidium bromide staining, DNA fragments were recovered from agarose gels and cloned into pMD‐18T (TaKaRa, Japan) according to the manufacturer's instructions, and ten positive clones were sequenced. The data were analyzed using quantification tool for methylation analysis ( QUMA) analyzer software.

### Animals

Female NOD‐scid and BALB/c (nu/nu) mice (Shanghai Jihui Laboratory Animal Care Co., Ltd.), aged 6–8 weeks (18–22 g), were housed individually under specific pathogen‐free conditions with adequate food and water and kept under a 12 h light‐dark cycle at 22–25 °C. After a 1‐week acclimation period, the NOD‐scid mice were given a subcutaneous injection of 2 × 10^6^ LM2 pCDH and pCDH‐HTR1A, MCF10 Ca1a pCDH and pCDH‐HTR1A cells into the right mouse mammary fat pad, while the BALB/c (nu/nu) mice were given an intravenous injection of 3 × 10^5^ MDA‐MB‐231 shScramble and shHTR1A cells. To explore the function of HTR1A on tumor metastasis in vivo, LM2 and MCF10 Ca1a stable overexpression cells, as well as 231 stable knockdown cells, were labeled with a retroviral construct expressing a green fluorescent protein/luciferase fusion protein. In addition, to assess the effect of HTR1A agonists combined with demethylation drugs such as 5‐aza‐2’‐deoxycytidine on breast cancers, another group of BALB/c (nu/nu) mice received a subcutaneous injection of 2 × 10^6^ LM2 cells. The xenograft models were randomly divided into different experimental and control groups as follows when the tumors reached 6–8 mm in diameter: a) control group (5% dimethyl sulfoxide (DMSO) + 40% PEG300 + 10% Tween 80 + ddH_2_O), b) vilazodone group, c) 5‐aza‐2’‐deoxycytidine group, and d) vilazodone + 5‐aza‐2′‐deoxycytidine group. All drugs were given at 5 mg kg^−1^ for 21 d.

All mice were euthanized by cervical dislocation and their tumors and lungs were harvested for subsequent experiments. The antitumor effects were determined by assessing the tumor volume, which was calculated twice a week using the following formula: tumor volume = 0.5 × length × width^2^. However, if weight loss exceeded 20% or a mouse became immobile and unable to intake food, euthanasia was performed. Nontumor‐related deaths were also excluded from the death records in the present analysis. All animal experiments conducted were approved and conducted in accordance with the Guide of the Institutional Animal Care and Use Committee of Fudan University Shanghai Cancer Center.

### Statistical Analysis

Differences between groups were evaluated by performing the unpaired *t*‐test. Each experiment was performed three times per group. The Kaplan–Meier method was used to calculate the cumulative survival time and differences in survival were analyzed with the log‐rank test. OS was defined as the interval between the start of the treatment and death. RFS was defined as the time from the day of surgery to the day of first recurrence or last follow‐up. Patients who died from other causes were considered to be censored with no event when calculating RFS. An unpaired *t*‐test was used to compare tumor growth between the groups. Data are presented as the mean ± SD (standard deviation). All data were analyzed with two‐tailed tests, with *p* <0.05 defining statistical significance. All statistical analyses were performed with statistical product and service solutions (SPSS) statistical software V19.0 (SPSS Inc., Chicago, IL, USA).

## Conflict of Interest

The authors declare no conflict of interest.

## Authors Contribution

Q.L. and H.S. contributed equally to this work. Q.Q.L. and H.F.S. contributed to the study design. X.L. contributed to data collection. X.L., Y.L., and B.J.X. performed statistical analysis, interpretation, and drafted the paper. W.J. and H.F.S. revised the paper. All authors contributed to critical revision of the final paper and approved the final version of the paper. W.J. supervised the study and provided financial support.

## Supporting information

Supporting Information

## Data Availability

The data that support the findings of this study are available from the corresponding author upon reasonable request.
