# Supplementary material for: HTR1A Inhibits the Progression of Triple‐Negative Breast Cancer via TGF‐β Canonical and Noncanonical Pathways
Source: Adv Sci (Weinh). 2022 Feb 24;9(12):2105672. doi: 10.1002/advs.202105672 (PMC9036047; doi:10.1002/advs.202105672)
Supplement: Supplementary file 1 — Supporting Information [file ADVS-9-2105672-s001.pdf]

ADVANCED SCIENCE
Open Access

Supporting Information

for *Adv. Sci.*, DOI 10.1002/advs.202105672

HTR1A Inhibits the Progression of Triple-Negative Breast Cancer via TGF-β Canonical and Noncanonical Pathways

Qiqi Liu, Hefen Sun, Yang Liu, Xuan Li, Baojin Xu, Liangdong Li and Wei Jin**

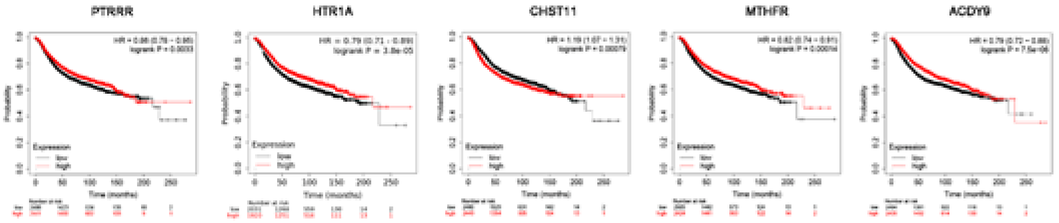

Supplementary Figure 1. The antitumor effect of HTR1A agonists.

The overall survival of indicated genes by the Kaplan-Meier plotter online database.

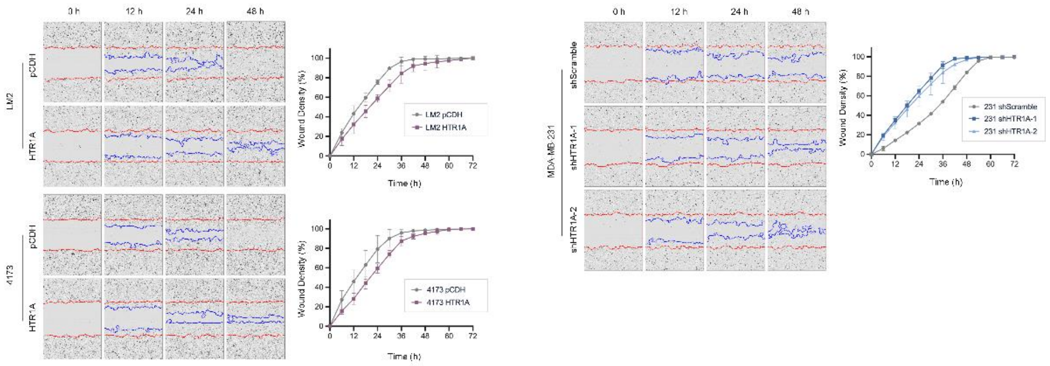

Supplementary Figure 2. HTR1A inhibited the migration of TNBC cells

The wound healing assay of HTR1A overexpression in LM2 and 4173 cells and HTR1A knockdown in MDA-MB-231 cells.

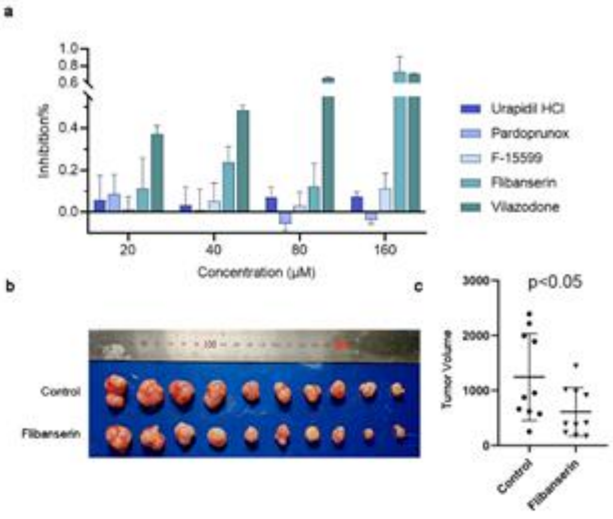

Supplementary Figure 3. The antitumor effect of HTR1A agonists.

a: The inhibition rate of HTR1A agonists in 48h on LM2 cells by CCK8; b-c: The tumor growth of mouse xenograft models treated with flibanserin and control.

Supplementary Table 1: The primer sequences of depression-related genes.

Gene	Primer sequence (5'-3')	Gene	Primer sequence (5'-3')
ACE	GGUGGUAUCUUCGAACCAATT	ITPR1	GGACGAGGCUGGAAAUGAATT
	GGUCCAACAUCUAUGACUUTT		GCACUUUGUUCACUGCAUATT
	CCAGGACUCAAGGUGACUUTT		GCUUUCAAUGUCUGCAAUATT
ADCY9	GCCAAGAAUCAUAGCCGAUTT	KLHL29	CCUGAUUGCACACUCCUAUTT
	CCAUCAGGAUGGUGUUCUUTT		GGAACCCGCAGAACAACAATT
	CCAAGCUGCUGUACGACAUTT		GCGUCGUGAUAAAGAAAUATT
APOE	GUCACAUUCCUGGCAGGAUTT	LHFPL2	CCGAGCUCAUUGCCUUCAUTT

	GAGUUGAAGGCCUACAAAUTT<br>CGUUGCUGGUCACAUUCCUTT		GCCAUAGACUACUGUGGACTT<br>GCAGAAAUUGCAACCUCUATT
CHST11	CCACUUGCUUGGGAUCCUUTT<br>GGUACGGCACCAAGAUCAUTT<br>GCAAAGUCUACGAGAACUATT	MAOA	GCUGAACCAUGAACAUUAUTT<br>GCAGCUAGGGAGGUCUUAATT<br>GCUUUACAUCCAGUGCAUUTT
DNAJB2	GGAGAUUUACGACCGCUAUTT<br>GCAGCUGAAGUCAGUCACATT<br>CCUACAGCCUGUCAGAGAUTT	MTHFR	GCACAUCCGAAGUGAGUUUTT<br>GCCAAUCAAAGACAACGAUTT<br>CCGGGUUAAUUACCACCUUTT
DRD4	GCCUCCAUCUUCAACCUGUTT<br>GACUACGUGGUCUACUCGUTT<br>CCUUCUUCGUGGUGCACAUTT	PCLO	GCCGAUUCCUAAGCAGCAATT<br>GCAACAAACUUUGUACCAUTT<br>GCAAGAAUCAAGUGUGUAATT
EHD3	CCUUUGGCAACGCCUUCUUTT<br>GGAAGGACAACAAGAAGAATT<br>CCAUGUACGACGAGAUCUUTT	PHACTR3	GGAGACGCUGACUUCAGAATT<br>GCGCUGAACGACUCCAUUATT<br>GCAAGCACUUGACAAGAUUTT
FREM3	GCUUUCAUGGUGGUGGUAATT<br>GCCCUUCAUCGUAUAUGAATT<br>GCCAGGUCCUGAUUAUCAA	PTPRR	GCUGAACAUAACCUUGCUUTT<br>CCAACGUAUCUCUUACAUUTT<br>CCAGUGAGCAGUAUGAAUU

	TT		TT
GNB3	GGGCAGCAGAAGACUGUAUTT<br>GGUCAUGACCUGUGCCUAUTT<br>CCUGGAUGACAACAAUAUUTT	SLC6A3	UCAACUCCCUGACGAGCUUTT<br>UCAUCUACCCGGAAGCCAUTT<br>CCAUCUAUGCGGCCUACAATT
HS6ST3	GCAGCCUUGUAGCUGCAAATT<br>GGCUGCUAUAACUUGACUUTT<br>GCAUUGAGGAUCUAAACUUTT	SLC6A4	CCACCGAAAUGGAUGCAUUTT<br>GCAUCUGGAAAGGCGUCAATT<br>CCUCCUCUUCAUCACGUAUTT
HTR1A	GCGGCAACACUACUGGUAUTT<br>GGCUUAUUGGCUUCCUCAUTT<br>GCAACUCCAAAGAGCACUUTT	SLC25A21	CCAAGCCUGAAGUCAGCUUTT<br>GCCAUUGCUGGAUUGGGAUTT<br>GGAGAGAUCAAGUACAGAATT
HTR1B	GGGUUCCUCAAGCCAACUUTT<br>CCUCGGUCACCUCUAUUAATT<br>CCAUGUCCAAUGAGGACUUTT	TPH2	GCAGCUCAACACUAAAUAATT<br>CCAUGAACUCUUGGGACAUTT<br>GCUUACAGGAAUGCCUUAUTT
HTR2A	GCUCUACAGUAAUGACUUUTT<br>CCAUGCCAAUACCAGUCUUTT<br>GCUUUGGCCUACAAGUCUATT	UGT2A1	GCUGUUCUCCCUUCAGAUATT<br>GCUUUAGAUGGUAGCCAUUTT<br>GCAUAAUGUGACUGUCCUATT
HTR3B	GCGUGCAGUUUAGAGACAUTT	VGLL4	GACCUGUUGAACUAUCAGUTT

	GCUGAUUCCUAGCAUCUUU TT		CCACGUCUCCAAAAUGAGU TT
	GCUUUGACCGACUGCUCUU TT		GCCUCAGUUACCAAAGAAA TT
NC-1	UUCUCCGAACGUGUCACGU TT	NC-2	GCGACGAUCUGCCUAAGAU

Supplementary Table 2. All antibodies used in this study.

Antibody	Species	Working concentration	Company
HTR1A	Rabbit	1:1000 for WB, 1:50 for IP	Abcam
TβRII	Mouse	1:2000 for WB, 1:50 for IP	Proteintech
p-Smad3	Rabbit	1:1000 for WB	Abcam
TβR1	Rabbit	1:1000 for WB	Abcam
Smad3	Rabbit	1:1000 for WB	Abcam
Total MEK	Rabbit	1:1000 for WB	CST
Phospho-MEK	Rabbit	1:1000 for WB	CST
Total ERK	Rabbit	1:1000 for WB	Abcam
Phospho-ERK	Rabbit	1:1000 for WB	Abcam
c-Myc	Rabbit	1:1000 for WB	Abcam
E-cadherin	Rabbit	1:1000 for WB	Proteintech
N-cadherin	Rabbit	1:25000 for WB	Proteintech
Fibronectin 1	Mouse	1:1000 for WB	Proteintech
TRIM21	Rabbit	1:1000 for WB, 1:200 for IP	Proteintech
PSMD7	Rabbit	1:1000 for WB, 1:200 for IP	Proteintech

Supplementary Table 3. The proteins detected by MS/MS.

Gene Symbol	Full Name	Protein score	Coverage (%)	Peptides	Unique	Avg. Mass
PPP1CC	Serine/threonine-protein phosphatase PP1-gamma catalytic subunit	173.04	37	12	1	36984
RPLP0	60S acidic ribosomal protein P0	163.02	49	11	11	34274
PAWR	PRKC apoptosis WT1 regulator protein	151.84	34	9	9	36568
EEF1A1	Elongation factor 1-alpha 1	148.29	27	9	9	50141

HNRNPD	Heterogeneous nuclear ribonucleoprotein D0	136.46	22	8	8	38434
PSMD7	26S proteasome non-ATPase regulatory subunit 7	123.42	13	5	5	37025
EIF3F	Eukaryotic translation initiation factor 3 subunit F	113.71	14	4	4	37564
HNRNPH3	Heterogeneous nuclear ribonucleoprotein H3	109.8	15	5	5	36926
TRIM21	E3 ubiquitin-protein ligase TRIM21	101.56	17	8	8	54170
JUN	Transcription factor AP-1	90.88	11	3	3	35676
CREB1	Cyclic AMP-responsive element-binding protein 1	83.3	9	3	3	36688
SEC13	Protein SEC13 homolog	79.76	17	4	4	35541
HNRNPA1L2	Heterogeneous nuclear ribonucleoprotein A1-like 2	78.76	9	4	1	34225
CORO1C	Coronin-1C	77.91	8	3	3	53249
EIF3G	Eukaryotic translation initiation factor 3 subunit G	68.07	11	3	3	35611
PLAUR	Urokinase plasminogen activator surface receptor	66.21	10	3	3	36978
WDR77	Methylosome protein 50	63.31	8	2	2	36724
MFGE8	Lactadherin	61.92	9	3	3	43105
CEBPB	CCAAT/enhancer-binding protein beta	58.7	7	1	1	36106
PLAUR	Urokinase plasminogen activator surface receptor	56.51	4	1	1	36978
